# Drug-eluting beads bronchial arterial chemoembolization vs. conventional bronchial arterial chemoembolization in the treatment of advanced non-small cell lung cancer

**DOI:** 10.3389/fmed.2023.1201468

**Published:** 2023-08-03

**Authors:** Guocan Yu, Yanqin Shen, Liangliang Chen, Xudong Xu, Jun Yang

**Affiliations:** ^1^Department of Thoracic Surgery, Affiliated Hangzhou Chest Hospital, Zhejiang University School of Medicine, Hangzhou, Zhejiang, China; ^2^Department of Nursing, Affiliated Hangzhou Chest Hospital, Zhejiang University School of Medicine, Hangzhou, Zhejiang, China

**Keywords:** NSCLC, DEB-BACE, BACE, objective response rate, disease control rate, PFS, os, safety

## Abstract

**Purpose:**

To compare the effectiveness and safety of drug-eluting bead bronchial artery chemoembolization (DEB-BACE) with conventional bronchial artery chemoembolization (cBACE) and provide a novel treatment option for advanced non-small cell lung cancer (NSCLC).

**Methods:**

Patients with advanced NSCLC underwent DEB-BACE or cBACE and were screened retrospectively. Progression-free survival (PFS) and overall survival (OS) were the primary outcome indicators, while technical success rate, objective response rate (ORR), disease control rate (DCR), and adverse events (AEs) were the secondary ones.

**Results:**

A total of 41 patients were enrolled in the study, 12 in the DEB-BACE group and 29 in the cBACE group, according to the treatment regimen. No patient achieved complete response. Eighteen patients achieved partial response (9 in each group), 15 patients achieved stable disease (3 in the DEB-BACE group and 12 in the cBACE group), and eight patients achieved progressive disease (all in the cBACE group) when treated for 2 months. The overall ORR and DCR were 43.9% (18/41) and 80.5% (33/41), respectively. ORR and DCR in the DEB-BACE group were 50.0% (9/12) and 100.0% (12/12), respectively, while ORR and DCR in the cBACE group were 31.0% (9/29) and 72.4% (21/29), respectively. Compared to cBACE, the ORR and DCR of DEB-BACE were significantly improved (*p* < 0.05). The median PFS was better in the DEB-BACE group than in the cBACE group (6.95 months vs. 3.20 months, respectively, Hazard Ratio [HR] = 0.416; *p* = 0.005). Furthermore, the median OS was significantly better in the DEB-BACE group than in the cBACE group (28.5 months vs. 22.5 months, respectively, HR = 0.316; *p* = 0.020).

**Conclusion:**

DEB-BACE has a good safety and therapeutic profile in advanced NSCLC and is superior to cBACE. DEB-BACE can be used as an alternative treatment option for advanced NSCLC, even in elderly patients.

## Introduction

Lung cancer is a common malignancy with a poor prognosis that poses a serious threat to public health (1). Non-small cell lung cancer (NSCLC) accounts for approximately 85% of all primary lung cancers (2). The efficacy of conventional chemotherapy combined/sequential radiotherapy is still limited for patients with inoperable advanced NSCLC (3). The scope of the application of gene-targeted therapy and immunotherapy is still relatively limited. Consequently, these factors are limiting the application of these therapies in clinical practice.

Angiogenesis is an important factor in the growth and proliferation of tumors (4). Vascular interventions are widely used in solid tumors with satisfactory effects, while at the same time reducing adverse effects (AEs) and improving patients’ quality of life (5). Bronchial arterial infusion (BAI) has been found to inhibit the growth of lung cancer cells, and it has been shown to be more effective than systemic chemotherapy (6). However, recent advancements in bronchial artery interventions have underlined the advantages of using conventional bronchial artery chemoembolization (cBACE) in all types of types of cancer, largely replacing BAI alone (7). cBACE is applied under low conditions and can benefit different types of lung cancer (8).

Drug-eluting beads (DEBs) are the most prominent materials used for embolization thanks to the recent advances in materials science. The use of DEBs loaded with chemotherapeutic agents for BACE is known as drug-eluting bead bronchial artery chemoembolization (DEB-BACE), which has been used in different types of advanced lung cancer with good results (9, 10). However, the use of DEB-BACE in lung cancer is still limited and even less studied compared to conventional cBACE (11). Therefore, the aim of this study was to compare the effectiveness and safety of DEB-BACE with cBACE in advanced NSCLC and justify the use of this novel treatment option for patients with advanced NSCLC.

## Materials and methods

### Participants

Patients with advanced NSCLC who were hospitalized at the Affiliated Hangzhou Chest Hospital, Zhejiang University School of Medicine, China between April 2019 and December 2022 and underwent DEB-BACE or cBACE were retrospectively enrolled. Inclusion criteria were: (a) clear NSCLC on pathological testing; (b) clinicopathological stage III-VI; (c) age > 18 years; (d) treatment with DEB-BACE or cBACE complete follow-up data. Patients were excluded if they had: (a) undergone BAI alone or bronchial artery embolization (BAE) alone; (b) not underwent DEB-BACE or cBACE incomplete treatment or follow-up information. The study protocol was approved by the Human Research Ethics Committee of the Affiliated Hangzhou Chest Hospital, Zhejiang University School of Medicine, China, and informed consent was obtained from patients or their guardians. Our study complies with the Declaration of Helsinki. Patient-related data were extracted from the medical system.

### Intervention

DEB-BACE as a therapeutic intervention.

### Control intervention

cBACE was the control intervention treatment.

### Outcomes

Progression-free survival (PFS) and overall survival (OS) were the primary outcome indicators, and technical success rate, objective response rate (ORR), disease control rate (DCR), and AEs were the secondary outcome indicators in the present study. PFS was defined as the periods from the start of the first DEB-BACE or cBACE treatment to tumor progression or death from any cause. OS was defined as the periods from the start of the first treatment to death from any cause. Furthermore, the technical success rate was defined as the number of patients who successfully completed DEB-BACE or cBACE as a proportion of all patients who needed to complete this treatment. Response evaluation criteria in solid tumors (RECIST 1.1) were used to evaluate the local response of the treated lung tumors using enhanced lung CT images (12). Complete response (CR) was defined as the complete disappearance of lung cancer lesions where all pathological lymph nodes were reduced to less than 10 mm in the short diameter, and partial response (PR) was defined as the sum of lung cancer lesion diameters that were reduced by at least 30% from baseline levels. Progressive disease (PD) was determined as the sum of diameters increases by more than 20% relative to the baseline (the sum of the smallest diameters measured before treatment), and the sum of diameters increases by at least 5 mm, or with the appearance of new lesions. Stable disease (SD), where the change in lesion diameter intervenes between PR and PD, with ORR being (CR + PR)/(CR + PR + SD + PD) × 100% and DCR being (CR + PR + SD)/(CR + PR+ SD + PD) × 100%. AEs are defined as a complication resulting from DEB-BACE or BACE treatment and commonly includes chest discomfort, nausea and vomiting, and fever. The local response of the treated lung tumors was evaluated after 2 months of treatment.

### Study design

A retrospective study was conducted to evaluate the efficacy and safety of DEB-BACE and cBACE in advanced NSCLC.

### Intervention procedure

**Figure 1:**
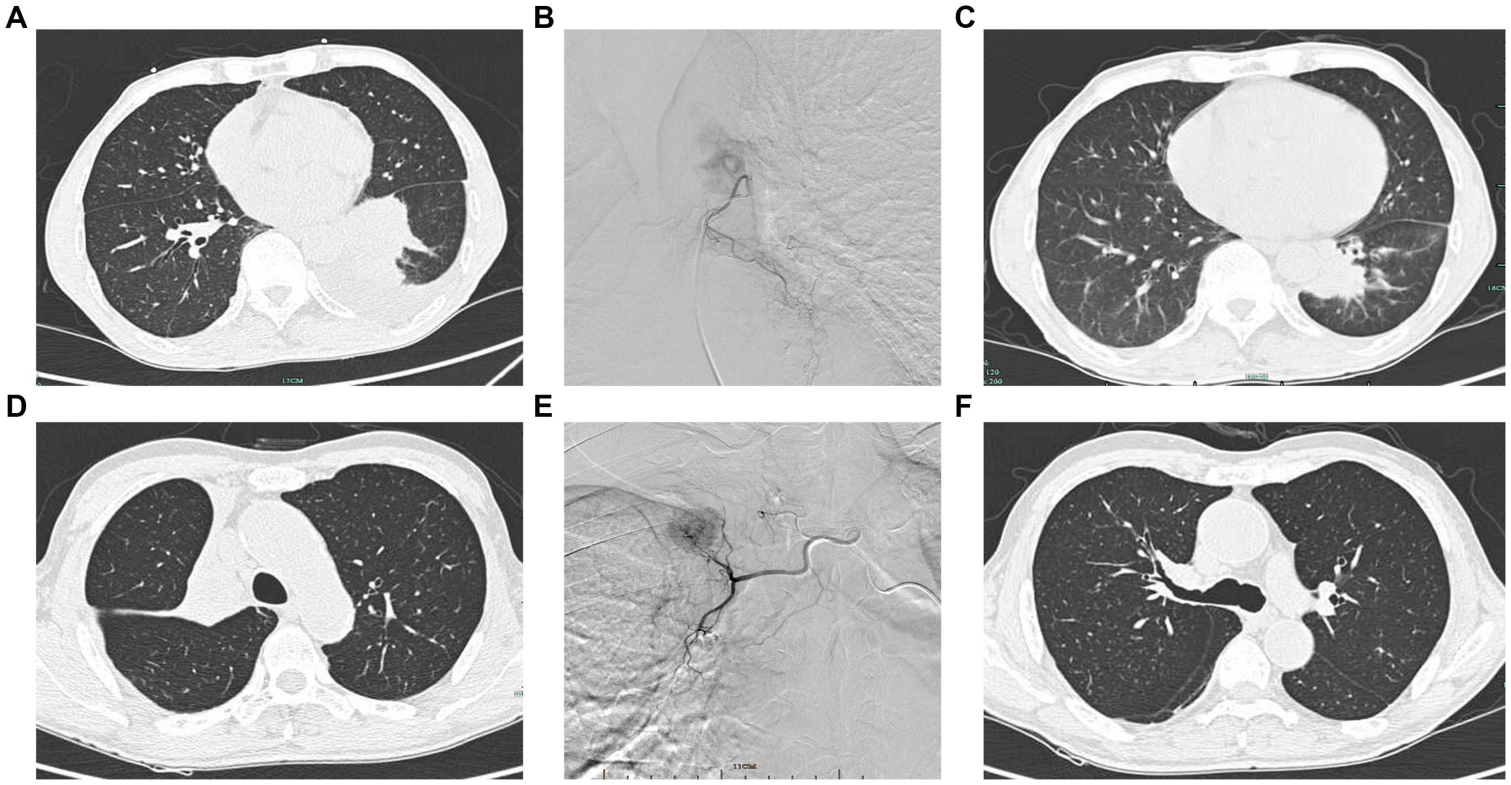
Interventional procedure images and computed tomography images before and after intervention. **(A)** Pre-interventional image of a case of squamous carcinoma of the left lower lung. **(B)** DSA showing left bronchial artery supplying left lower lung cancer. **(C)** Significant shrinkage of the left lower lung mass after intervention. **(D)** Squamous carcinoma of the right upper lobe opening with right upper lung atelectasis. **(E)** DSA showing right bronchial artery supplying the right upper lung mass. **(F)** Regression of the tumor of the right upper lobe opening and lung reopening after intervention.

The treatment choice solely depended on patients and their guardians after fully informing them of the treatment options available, including the pros and cons of the relevant options. Prior to the intervention, all patients underwent Computed Tomography Angiography (CTA) of the bronchial arteries to identify tumor trophoblastic vessels and guide the localization of the relevant vessels intraoperatively. Both DEB-BACE and cBACE were performed under digital subtraction angiography (DSA) (Figure 1). The BAEC or DEB-BACE treatment were conducted once every 3 weeks.

Patients were placed in the supine position, lying flat on the DSA, and the right femoral artery was the preferred site for arterial puncture. A 4F-Corber catheter (Cordis, FL, United States) was placed through the catheter sheath. The tumor-supporting blood vessels were located under the guidance of preoperative CTA of the bronchial arteries, and a 2.7F microcatheter (Merit Medica, United States) was selectively cannulated into the relevant vessels confirmed by DSA angiography. In principle, it is necessary to fully expose the tumor and identify all possible blood supply vessels. When a spinal artery was suspected, a microcatheter should be used for superselection to protect the spinal artery.

Drug infusion and embolization were performed after confirming the tumor target vessel and suitably positioning the microcatheter. In the cBACE group, docetaxel (Hengrui Pharmaceutical Co., ltd, Jiangsu, China) combined with cisplatin (Haosen Pharmaceutical Group Co., ltd, Jiangsu, China) was used for squamous cell carcinoma (SCC), while gemcitabine (Haosen Pharmaceutical Group Co., ltd, Jiangsu, China) combined with cisplatin for adenocarcinoma (ADC). The chemotherapeutic drugs were dissolved in 50 mL of saline separately, and docetaxel or gemcitabine solution was injected slowly into the target vessel through the catheter first, followed by cisplatin for at least 10 min for each drug. After the injection of chemotherapeutic drugs, the target vessel was embolized using 100–200 μm microspheres (Merit Medica, United States) that were not loaded with the drug. The endpoint of embolization was flow arrest or near arrest, and successful embolization was confirmed by re-imaging after embolization. After the procedure, the puncture site was sand-bagged with pressure for 6 h and the punctured leg was immobilized for 24 h. Chemotherapeutic drug doses were determined according to the body surface area, typically 75 mg/m^2^ for docetaxel, 1,000 mg/m^2^ for gemcitabine, and 75 mg/m^2^ for cisplatin.

In the DEB-BACE group, embolization was performed using HepaSphere (50–100 μm; Merit Medica, United States) DEB loaded with cisplatin. Previous study had demonstrated that HepaSphere can effectively load cisplatin and release it in a stable manner (13, 14). In this study, the HepaSphere DEB loaded with 30 mg cisplatin was prepared by the operator using the 2× method as follows (13): 20–30 min before the procedure, 2 mL of 10% NaCl and 8 mL of non-ionic contrast agent were aspirated using a 10-ml syringe, and the mixture was added to the 30-mg cisplatin vial, fully dissolved and then withdrawn. The cisplatin vial was placed in hot water (30°C) and shaken to speed up the complete dissolution of cisplatin. Consequently, 10 mL of the above mixture were withdrawn with a syringe, and the mixture was added to the HepaSphere bottle. The HepaSphere bottle was then shaken gently, not violently from side to side, for approximately 10 min (the HepaSphere bottle can be repeatedly turned upside down during the waiting period) to bring the microspheres into full contact with the mixed solution. The liquid containing the microspheres in the bottle was then withdrawn using a syringe and transferred to the operating table via a tee.

Standard precautions were: do not use water for injection or glucose injection for preparation; do not remove the yellow plastic cap when opening the cap of the HepaSphere ampoule; it is recommended that the needle should be inserted into the rubber stopper of the ampoule at a 45° angle; a 1:1 mixture of contrast and saline, 10 mL each, is recommended for embolization before use. The pre-embolization bronchial artery perfusion chemotherapy drug was the same as in the cBACE group, except that the cisplatin dose was reduced by 30 mg.

### Statistical analysis

Excel 2019 was used to record patient information obtained from the electronic medical record system. SPSS 24.0 and GraphPad Prism 6.02 (GraphPad Software, Inc., CA, United States) were used for statistical analysis of the data. Differences between the two groups were compared using the *t*-test for measurement data and the Fisher’s exact test or chi-square test for count data. Survival curves were plotted in GraphPad Prism 6.02 using the Kaplan–Meier (KM) method, and the difference in survival between the two groups was tested using the log-rank (Mantel–Cox) test. A value of *p* < 0.05 was considered a statistically significant difference between the two sets.

## Results

### Patient clinical features

**Figure 2:**
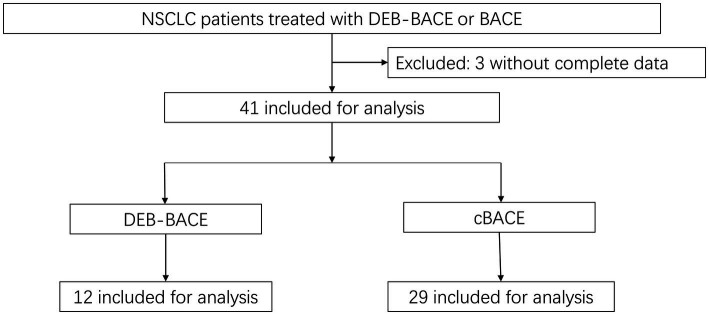
The screening process of included patients. DEB-BACE, drug-eluting beads bronchial arterial chemoembolization; cBACE, conventional bronchial arterial chemoembolization.

We initially screened 44 patients with NSCLC who underwent DEB-BACE or cBACE. Three patients lacked treatment or follow-up information and were thus excluded from the study, resulting in the inclusion of a total of 41 patients (35 males and six females), of whom 12 underwent DEB-BACE and 29 underwent cBACE. The patient screening process is illustrated in Figure 2. The mean age of all patients was 66.4 ± 9.1 years, the mean age of the DEB-BACE group was 66.9 ± 9.0 years and the mean age of the cBACE group was 66.1 ± 9.1 years. For non-first-line treatment patients, all had received standard treatment regimens for lung cancer. There was no significant difference in previous treatment regimens between the two groups. Differences in general clinical characteristics, including age, gender, smoking history, hemoptysis, concomitant diseases, size of tumor, pathology type, tumor stage, lines of treatment, times of interventions, and tumor site, between the two groups were non-significant (*p* > 0.05; Table 1). All patients were successfully treated with DEB-BACE or cBACE, and there were no patients with an unsuccessful procedure, with a success rate of 100%.

**Table 1:** Clinical characteristics of the included patients.

Characteristics	All (*n* = 41)	DEB-BACE (*n* = 12)	cBACE (*n* = 29)	*p* value
Age (years), mean ± SD	66.4 ± 9.1	66.9 ± 9.0	66.1 ± 9.1	0.799
**Gender**				
Male, *n* (%)	35 (85.4)	10 (83.3)	25 (86.2)	0.813
Female, *n* (%)	6 (14.6)	2 (16.7)	4 (13.8)	
Smoking history, *n* (%)	34 (82.9)	10 (83.3)	24 (82.8)	0.965
Hemoptysis, *n* (%)	14 (34.1)	6 (50.0)	8 (27.6)	0.168
**Concomitant diseases**				
Hypertension, *n* (%)	11 (26.8)	1 (8.3)	10 (34.5)	0.086
T2DM, *n* (%)	1 (2.4)	1 (8.3)	0 (0.0)	0.116
COPD, *n* (%)	4 (9.8)	1 (8.3)	3 (10.3)	0.843
Tuberculosis, *n* (%)	17 (41.5)	6 (50.0)	11 (37.9)	0.475
Pneumoconiosis, *n* (%)	1 (2.4)	0 (0.0)	1 (3.4)	0.515
Cerebral infarction, *n* (%)	1 (2.4)	0 (0.0)	1 (3.4)	0.515
**Pathology type**				
Size of tumor (cm)	6.3 ± 2.2	7.2 ± 1.8	5.9 ± 2.2	0.089
Squamous cell carcinoma, *n* (%)	36 (87.8)	10 (83.3)	26 (89.7)	0.574
Adenocarcinoma, *n* (%)	5 (12.2)	2 (16.7)	3 (10.3)	
**Tumor stage**				
III, *n* (%)	34 (82.9)	11 (91.7)	23 (79.3)	0.339
IV, *n* (%)	7 (17.1)	1 (8.3)	6 (20.7)	
**Lines of treatment**				
Frist line	29 (70.7)	9 (75.0)	20 (69.0)	0.699
Second line	6 (14.6)	2 (16.7)	4 (13.8)	0.813
Third or later line	6 (14.6)	1 (8.3)	5 (17.2)	0.463
Times of intervention	1.8 ± 0.7	1.8 ± 0.6	1.8 ± 0.7	1.000
**Tumor site**				
Left upper lung, *n* (%)	10 (24.4)	2 (16.7)	8 (27.6)	0.459
Left lower lung, *n* (%)	9 (22.0)	1 (8.3)	8 (27.6)	0.175
Right upper lung, *n* (%)	13 (31.7)	5 (41.7)	8 (27.6)	0.378
Right lower lung, *n* (%)	3 (7.3)	1 (8.3)	2 (6.9)	0.872
Right middle lung, *n* (%)	6 (14.6)	3 (25.0)	3 (10.3)	0.227

DEB-BACE, drug-eluting beads bronchial arterial chemoembolization; cBACE, conventional bronchial arterial chemoembolization; SD, standard deviation; T2DM, type 2 diabetes; COPD, chronic obstructive pulmonary disease.

### Local response of lung tumors

No patients achieved CR, while 18 patients (*n* = 9 in each group) achieved PR. Furthermore, 15 patients achieved SD (*n* = 3 in the DEB-BACE group and *n* = 12 in the cBACE group) and eight patients achieved PD (all in the cBACE group) when treated for 2 months. The overall ORR and DCR were 43.9% (18/41) and 80.5% (33/41), respectively. ORR and DCR in the DEB-BACE group were 50.0% (9/12) and 100.0% (12/12), respectively, while ORR and DCR in the cBACE group were 31.0% (9/29) and 72.4% (21/29), respectively. Compared to cBACE, the ORR and DCR of the DEB-BACE group were significantly improved (*p* < 0.05; Table 2).

**Table 2:** Local response of lung tumors after interventional therapy.

Parameter	All (*n* = 41)	DEB-BACE (*n* = 12)	cBACE (*n* = 29)	*p* value
CR, *n* (%)	0 (0.0)	0 (0.0)	0 (0.0)	–
PR, *n* (%)	18 (43.9)	9 (75.0)	9 (31.0)	0.010
SD, *n* (%)	15 (36.6)	3 (25.0)	12 (41.4)	0.322
PD, *n* (%)	8 (19.5)	0 (0.0)	8 (27.6)	0.043
ORR, (%)	43.9	75.0	31.0	0.010
DCR, (%)	80.5	100.0	72.4	0.043

DEB-BACE, drug-eluting beads bronchial arterial chemoembolization; cBACE, conventional bronchial arterial chemoembolization; CR, complete response; PR, partial response; SD, stable disease; PD, progressive disease; ORR, objective response rate; DCR, disease control rate.

### Survival status

**Figure 3:**
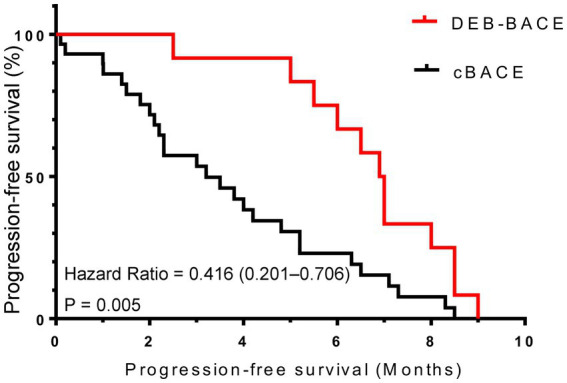
The survival curves for progression-free survival. DEB-BACE, drug-eluting beads bronchial arterial chemoembolization; cBACE, conventional bronchial arterial chemoembolization.

**Figure 4:**
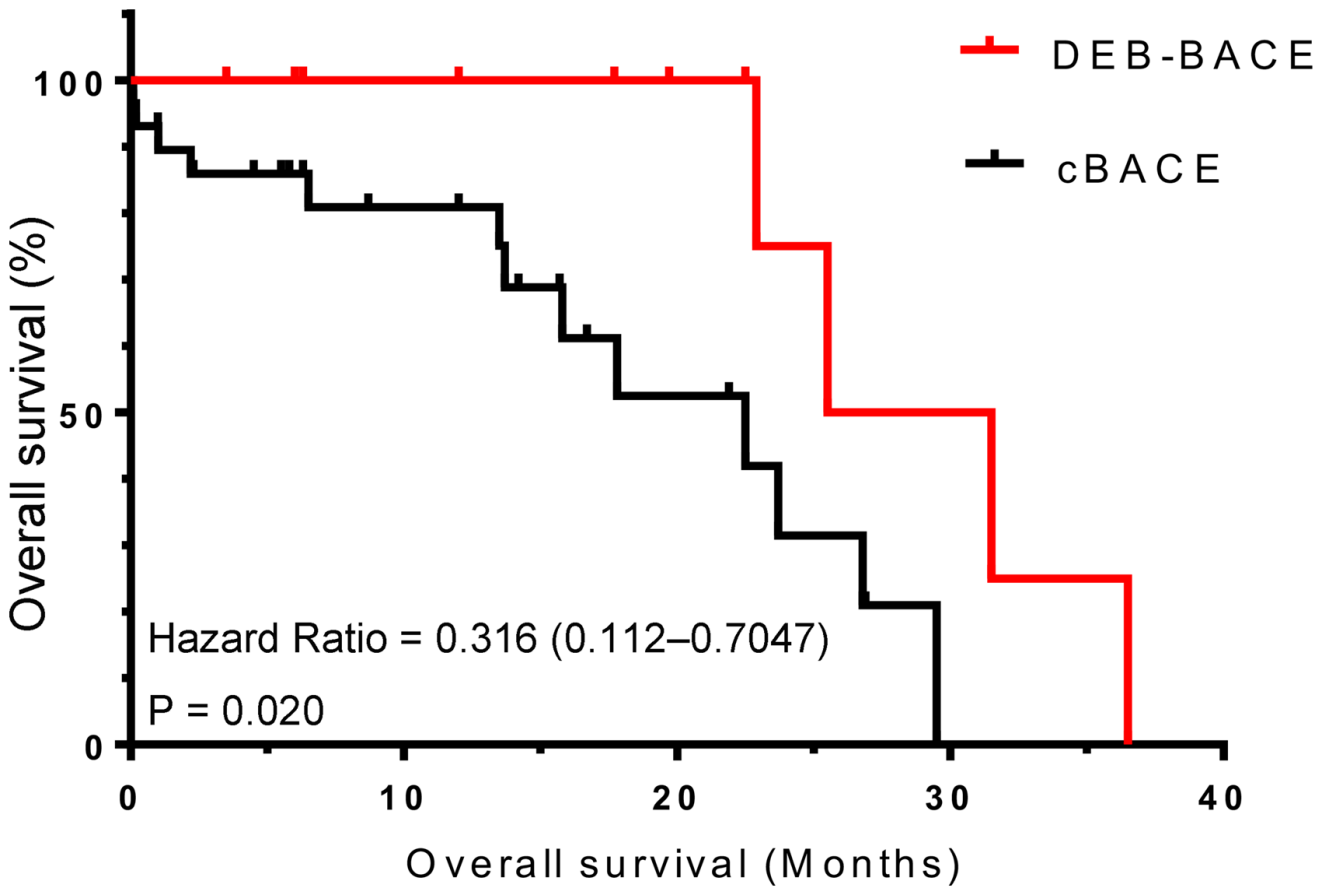
The survival curves for overall survival. DEB-BACE, drug-eluting beads bronchial arterial chemoembolization; cBACE, conventional bronchial arterial chemoembolization.

As of January 2023, the duration of follow-up for all patients ranged from 0.1 to 36.5 months, with a mean follow-up time of 13.7 ± 9.7 months. The median PFS was better in the DEB-BACE group than in the cBACE group (6.95 months vs. 3.20 months, respectively, Hazard Ratio [HR] = 0.416, 95% CI: 0.201–0.706; *p* = 0.005). The survival curves are shown in Figure 3. The median OS was significantly better in the DEB-BACE group than in the cBACE group (28.5 months vs. 22.5 months, respectively, HR = 0.316, 95% CI: 0.112–0.7047; *p* = 0.020). The survival curves are shown in Figure 4.

### AEs

Two cases of post-treatment hemoptysis resulting in death were noted in the cBACE group. One patient in the cBACE group had significant post-operative abdominal pain and was eventually diagnosed with bleeding from a metastatic abdominal lesion and died. No serious complications occurred in the remaining patients. However, fever, chest discomfort, nausea, and vomiting were common complications. The number of relevant complications is shown in Table 3, and the incidence of complications in both groups was not statistically significant (*p* > 0.05).

**Table 3:** The incidence of adverse reactions after interventional therapy.

Parameter	DEB-BACE (*n* = 12)	cBACE (*n* = 29)	*p* value
Fever, *n* (%)	2 (16.7)	6 (20.7)	0.767
Chest discomfort, *n* (%)	1 (8.3)	4 (13.8)	0.627
Abdominal pain, *n* (%)	1 (8.3)	2 (6.9)	0.872
Nausea and vomiting, *n* (%)	2 (16.7)	5 (17.2)	0.965
Hemoptysis, *n* (%)	0 (0.0)	2 (6.9)	0.351

DEB-BACE, drug-eluting beads bronchial arterial chemoembolization; cBACE, conventional bronchial arterial chemoembolization.

## Discussion

Effective treatment of advanced NSCLC remains challenging (15). Although traditional methods, such as intravenous systemic chemotherapy, radiotherapy, and combined radiotherapy, continue to be the standard of care, these treatments induce numerous systemic AEs and complications (16). In addition, these treatments are poorly tolerated by patients, especially those of advanced age, who find it difficult to cooperate well with treatment and whose quality of life is somewhat compromised. In contrast, the efficacy of chemotherapy seems to have reached a bottleneck, and further improvements are deemed difficult (16).

Transbronchial artery intervention is an alternative method for lung cancer treatment. Since Kahn et al. first reported BAI for lung cancer in 1965 (17), interventional treatment for lung cancer has developed considerably, both in terms of technology and materials. BAI significantly improved short-term outcomes for patients, reduced the incidence of AEs, and improved quality of life compared to systemic chemotherapy (6). BAI could also have advantages over radiotherapy (18). Currently, there are several transbronchial artery interventions, such as BACE, and DEB-BACE, which have shown good efficacy in both intermediate and advanced lung cancer (19). The growth and proliferation of lung cancer cells requires nutrients from the trophoblastic vessels of lung cancer, and the proliferation and growth of lung cancer is based on angiogenesis, which is the anatomical basis of endovascular interventions for lung cancer (20). Most researchers now agree that the pulmonary artery generally acts only as a smaller maintenance vessel in lung cancer, whereas the bronchial artery benefits more from a significantly stronger neovascularization capacity than the pulmonary artery and becomes the main blood supply vessel in lung cancer (21). Bronchial artery blood supply accounts for the vast majority of blood supply in central or larger lung cancers, and bronchial artery blood supply increases as lung cancer grows and proliferates, making it the main source of blood supply in lung cancer (22, 23).

BACE combines BAI with BAE, incorporating the advantages of both methods. On the one hand, embolization blocks the blood supply to cancer and cuts off the nutrient supply, causing tumor cells to be damaged and growth-inhibited due to ischemia and hypoxia. On the other hand, while BAI creates a high concentration of local drugs in lung cancer, the embolization of blood vessels reduces blood flow washout, which prolongs the residence time of local chemotherapeutic drugs in the vascular bed of cancer and the duration of drug action (24). This in turn leads to the inhibition of the transmembrane ion pump and uptake of more drugs, intensifying the chemotherapeutic effect (25). This dual effect renders BACE more advantageous than systemic chemotherapy and BAI alone due to its better therapeutic efficacy without the concomitant increase in treatment-related adverse effects (26). Even in chemotherapy-insensitive recurrent lung cancer, mechanical embolization of the bronchial arteries alone is equally effective (27). Theoretically, BACE can be performed in all types of lung cancer, and may be useful as long as the blood supply vessels are present, without the need for other relevant tests. In cases of intermediate to advanced lung cancer, especially central lung cancer, often combined with haemoptysis, and the risk of death is very high, affecting the patients’ prognosis (28). In addition to the usual hemostatic drugs, BAE is by far the most effective treatment for hemoptysis, based on the theory that this condition mainly originates from the bronchial arteries (29). In patients with intermediate to advanced lung cancer combined with hemoptysis, BACE is very appropriate to enhance the antitumor effect while stopping hemoptysis, with significant results for the purpose of etiological treatment (30).

DEB can release the contained drug within 600 μm of itself through ion exchange, which subsequently enters into the tumor target tissue rapidly. Furthermore, the loaded chemotherapeutic drug can be released slowly and continuously, maintaining the drug concentration inside the tumor at a high level for a longer period of time (31), coupled vascular embolism that slows blood flow and reduces the inflow of chemotherapeutic drugs into the circulation and low drug concentrations in non-target tissues. DEB can also maintain the release of high drug concentrations for up to 3 months after drug delivery. For these reasons, DEB is widely used in the interventional treatment of solid tumors, especially liver tumors (32). Several studies have shown that transarterial chemoembolization of liver tumors using DEB (DEB-TACE) is superior to conventional chemoembolization with iodinated oil in terms of both short-term and long-term efficacy (33, 34). As research progressed, the use of DEB in lung cancer, known as DEB-BACE, has increased, and Bie et al. (35) reported that the ORR and DCR of six patients treated with DEB-BACE for lung cancer was 50 and 100%, respectively. A study including 23 patients with unresectable lung cancer showed an overall efficacy rate of 78.3%, a DCR of 100%, and a median OS of 16.5 months, and no serious complications were observed (11). Some previous studies showed that DEB-BACE was more effective than intravenous systemic chemotherapy alone (36, 37). DEB-BACE is also more effective than cBACE in the treatment of intermediate to advanced lung cancer, with better ORR and PFS observed, and the safety profile of this treatment modality is very good (7). DEB-BACE has also demonstrated good clinical results in treating lung cancer patients with hemoptysis, with the dual purpose of hemostasis and tumor control (38). Some studies have also used DEB-BACE in the neoadjuvant treatment of SCC (39), with excellent results, and small cell lung cancer, with good results in terms of recurrent/refractory SCLC (40). These studies suggested that DEB-BACE in lung cancer can be an efficient approach that could achieve superior results.

DEB-BACE or cBACE is currently more commonly used in pulmonary SCC than in pulmonary ADC. The patients included in this study were also overwhelmingly SCC patients, and the tumor types were similar to the ones investigated in previous studies (41). The success rate of our interventions was 100%, demonstrating that the threshold for vascular interventions was not high. In addition, there was no restriction on the type of lung cancer, and thus treatment can be carried out for a wide range of applications as long as the preoperative CTA of bronchial arteries indicates the presence of tumor trophoblastic vessels. Our findings suggested that DEB-BACE was superior to cBACE in advanced NSCLC, resulting in better ORR, DCR, and longer survival. Both ORR and DCR after 2 months of intervention in this study were satisfactory. It should be mentioned, that the efficacy of this intervention after 6 months of treatment was not evaluated because the vast majority of patients in this study underwent first-line interventions, while some patients were treated with other treatment modalities as their disease progressed during the follow-up period, and the subsequent local tumor response was not due to the intervention alone. Previous relevant studies have also not included patients undergoing first-line interventions and only had a role in interventional treatment during the assessment cycle. PFS was consistent with the findings of other relevant studies, suggesting that this intervention provides similar results in terms of local tumor control, regardless of the treatment lines. Certainly, DEB-BACE was significantly more effective than cBACE, suggesting that DEB-BACE was the superior choice. The OS observed in our study (either in the DEB-BACE or cBACE groups) was significantly better than that found in other relevant studies, probably because previous studies included more patients with VI, whereas the proportion of VI patients in our study was lower. More specifically, previous studies included patients with treatment-failed or refractory lung cancer, whereas most of the patients included in our study were first-line-treated after failure of relevant interventional therapy, followed by other antitumor treatments that produced some efficacy. These factors contributed to the longer survival rates of patients in the present study, which demonstrated superior OS. OS was significantly longer in the DEB-BACE group than in the cBACE one, indicating that DEB-BACE was more effective in advanced NSCLC. These results may also suggest that the benefit of vascular intervention is greater in first-line treated patients or in patients at an earlier tumor stage. However, the number of patients included in this study was limited, and the associated bias may be significant. Therefore, our results should be interpreted with caution.

There may be differences in the effectiveness of DEB-BACE treatment using different drugs or different DEBs. A study focused on evaluating the efficacy of gemcitabine-loaded callispheres DEB in patients with advanced lung cancer demonstrated median PFS of 8.8 months and median OS 10.0 months (42). The median PFS of our study was inferior to that study, but the median OS was superior. The different types of lung cancer, different tumor stages, different lines of treatment and different regimens in the two studies may have contributed to the different results. However, the sample sizes of both studies were limited and no randomized controlled studies were performed, and these results need to be treated with caution.

AEs of DEB-BACE or cBACE mainly comprised complications due to vascular embolism and chemotherapeutic agents. The majority of patients included in the present study were older than 60 years of age and were well tolerated overall. The overall incidence of AEs in this study was low, and no AEs, such as spinal artery injury due to ectopic embolism and severe myelosuppression, occurred. One patient died 3 days postoperatively from hemorrhage due to abdominal metastasis, which was not caused by cBACE itself. Two patients in the cBACE group had massive hemoptysis approximately 1 month post-intervention, leading to death. Both patients had central SCC, and lung CT at the time of hemoptysis suggested an improving lung lesion with a large central area of necrosis followed by cavitation. Therefore, it is essential to be alert to the possibility of hemoptysis in central SCC should cavitation develops after treatment and undergo relevant guidance and education. The results of the present study suggested a good safety profile for DEB-BACE or cBACE in lung cancer applications, even in elderly patients.

The present study inevitably has some shortcomings. First, the number of patients included was limited, especially in the DEB-BACE group, increasing the bias of the presented findings. Second, this study was a retrospective study that patient selection might have also been biased because the enrollment was not randomized. Third, different tumor types may have led to differences in treatment outcomes. Therefore, randomized controlled studies with large patent samples are needed to further clarify the effects of these treatments and the differences of their effects in different tumor types.

## Conclusion

DEB-BACE had a good safety and therapeutic profile in advanced NSCLC and was superior to cBACE. DEB-BACE can be used as an alternative treatment option for advanced NSCLC, even in elderly patients.

## Data availability statement

The original contributions presented in the study are included in the article/supplementary material, further inquiries can be directed to the corresponding authors.

## Ethics statement

The studies involving human participants were reviewed and approved by The Human Research Ethics Committee of the Affiliated Hangzhou Chest Hospital, Zhejiang University School of Medicine. The patients/participants provided their written informed consent to participate in this study.

## Author contributions

XX and JY: conceptualization and project administration. XX, JY, and YS: methodology. GY and LC: software. GY, YS, and XX: validation. GY and YS: formal analysis. YS and LC: investigation. XX: resources. GY and JY: data curation and visualization. GY: writing—original draft preparation. GY and XX: writing—review and editing. JY: supervision. GY: funding acquisition. All authors contributed to the article and approved the submitted version.

## Funding

This work was supported by GY (20201203B183), Hangzhou Science and Technology Bureau, http://kj.hangzhou.gov.cn. The funder has and will not have a role in study design, data collection and analysis, decision to publish, or preparation of the manuscript.

## Conflict of interest

The authors declare that the research was conducted in the absence of any commercial or financial relationships that could be construed as a potential conflict of interest.

## Publisher’s note

All claims expressed in this article are solely those of the authors and do not necessarily represent those of their affiliated organizations, or those of the publisher, the editors and the reviewers. Any product that may be evaluated in this article, or claim that may be made by its manufacturer, is not guaranteed or endorsed by the publisher.
